# A one-year unisexual *Schistosoma mansoni* infection causes pathologic organ alterations and persistent non-polarized T cell-mediated inflammation in mice

**DOI:** 10.3389/fimmu.2022.1010932

**Published:** 2022-11-24

**Authors:** Martina Sombetzki, Cindy Reinholdt, Franziska Winkelmann, Anne Rabes, Nicole Koslowski, Emil C. Reisinger

**Affiliations:** Department of Tropical Medicine, Infectious Diseases and Nephrology, University Medical Center Rostock, Rostock, Germany

**Keywords:** unisexual infection, non-polarzzed T-cell response, schistosoma mansoni, hepatitis (general), longterm infection

## Abstract

In exhibiting gonochorism and phenotypic sexual dimorphism, *Schistosoma* spp. are unique among trematodes. Only females mating with male schistosomes can produce the highly immunogenic parasite eggs which determine the clinical picture of the disease schistosomiasis. The strong immune-modulatory effect of the eggs masks the influence of the adult worms. To shed light on the complexity of the immune response triggered by adult worms of *Schistosoma mansoni*, we performed a long-term unisexual infection experiment in mice. We were able to demonstrate that both male and female schistosomes can survive unpaired for one year in the murine host. Furthermore, unisexual *S. mansoni* infection leads to pronounced inflammation of the liver characterized by a non-polarized Th1/Th2 immune response, regardless of worm sex.

## Introduction

Despite decades of eradication efforts, schistosomiasis remains a challenging global health problem. Over 200 million people worldwide are affected and the condition is responsible for an estimated 1.9 million disability-adjusted life years (DALYs) (1–3). Schistosomiasis is spread by digenetic blood flukes of the genus *Schistosoma* (*S.*) spp., which is endemic in approximately 78 countries, predominantly in the tropics and subtropics (4). The three main species responsible for the disease in humans are *S. mansoni*, *S. haematobium* and *S. japonicum*, though most experimental studies focus on *S. mansoni*, one of the two species which cause intestinal schistosomiasis. Since no vaccine exists against these multicellular organisms, treatment options are mainly based on the anthelmintic praziquantel (PZQ). PZQ kills the adult worms in the host but unfortunately does not protect against infection. Furthermore, large-scale deworming efforts in endemic areas could promote the emergence of PZQ-resistant strains (5).

Parasitic infection with schistosomes does not go unnoticed by the host immune system, although schistosomes have developed strategies to protect themselves from the host’s immune defenses (6, 7). Infection and contact to worm antigens cause a weak transient Th1 response to occur which is characterized by the production of interferon-γ (IFN-γ) and tumor necrosis factor-α (TNF-α), among other signaling proteins. After worms reach sexual maturity 4-5 weeks post-infection, egg production begins. The transient Th1 response is now replaced by a dominant persistent Th2 response. Egg antigens are recognized as foreign by the host immune system and trigger a strong perioval granulomatous immune response (8). Granuloma formation is caused by a type IV T cell-mediated hypersensitivity reaction (9). When CD4^+^ T cells come into contact with egg antigens, cytokines (IFN-γ, TNF-α, and IL-5) are released which cause eosinophils and endothelial cells to become activated. Proteomic analyses of egg excretory/secretory products have shown that highly immunogenic proteins are secreted abundantly. These include IPSE (IL-4-inducing principle of schistosome eggs (also known as alpha-1 and *S. mansoni* chemokine binding protein, SmCKBP) and a ribonuclease omega-1. IPSE and omega-1 have been shown to promote Th2 differentiation directly (10–13). Secretion of the Th2 cytokines IL-13 and IL-4 activates hepatic stellate cells, which now massively produce extracellular matrix (collagen), leading in turn to liver fibrosis (Symmers’s pipe stem fibrosis) (14).

Clearly, the parasite eggs are highly immunogenic and determine the clinical picture of the disease. In addition, however, their strong immune modulatory effects might mask the influence of the worms themselves. To better understand the complexity of the immune response triggered by *S. mansoni* infection it is important to consider the host immune response to the worms alone. Studies to have done so (15–18) use infection models in which the final hosts are infected exclusively with male or female schistosomes and no oviposition occurs. Within these studies, male worms have been shown to affect the immune response *via* antigen production or *via* inflammatory processes (16, 19). Single male worms produce more antigens than single female worms, causing a stronger activation of the immune system and thus a stronger inflammatory response (16). An infection with exclusively male schistosomes causes a higher spleen weight in the final host and a higher number of leukocytes in the blood than an infection with exclusively female schistosomes (16). Conversely, in a study considering the effect of re-infection following unisexual infection (18), we were able to show that an 11-week unisexual pre-infection with female schistosomes suppresses the Th2 response upon subsequent re-infection with bisexual schistosomes and upregulates the expression of cytotoxic T-lymphocyte-associated protein 4 (CTLA-4). This protein is expressed on regulatory T cells and could be an explanation for the reduced hepatic fibrosis observed in mice pre-infected with female schistosomes. In another study by our research group, the suppression by female schistosomes of the host immune response was studied in more depth using the “air pouch model”. It was shown that the number of recruited inflammatory cells was significantly lower in the group pre-infected with female schistosomes than in the group pre-infected with male schistosomes, as were cytokine and chemokine levels (18, 20, 21).

Because of this distinctly sex-specific immune response elicited by adult worms, the goal of this study was to determine, first, how long worms of the different sexes survive unpaired in the host and, second, what immunological effects long-term unisexual infection causes in the host.

## Methods

### Long-term unisexual infection with *Schistosoma mansoni* in mice

The *Schistosoma mansoni* (*S. mansoni*, Belo Horizonte strain) life cycle was maintained using *Biomphalaria glabrata* (*B. glabrata*) freshwater snails as intermediate hosts and 6- to 8-week-old female NMRI mice as definitive hosts, as previously described (22). To obtain cercariae for the subsequent unisexual infection of mice, *B. glabrata* snails were exposed to individual *S. mansoni* miracidia, and cercariae were harvested 6 weeks later. The sex of the cercariae was determined by DNA amplification of sex-related chromosome segments using female-specific primers, as previously described (18). The following study design was used: 6- to 8-week-old female NMRI mice were percutaneously infected with 300 male (m, n=6) or female (f, n=5) *S. mansoni* cercariae. Naive control mice were not infected (n, n=4). Mice were sacrificed by cervical dislocation 52 weeks after infection under isoflurane anesthesia.

### Macroscopic and microscopic evaluation of organs and serum biochemistry

At 52 weeks post-infection, the collected livers and spleens were weighed, and the ratio of liver or spleen weight to body weight was determined. Serum biochemistry for alanine aminotransferase (ALT), aspartate aminotransferase (AST) and alkaline phosphatase (AP) was performed using UniCel^®^ DxC 800 Synchron^®^ Clinical System (Beckman Coulter GmbH). For histological evaluation, half of the right liver lobe was fixed in 4% neutral buffered formalin solution (Merck, Darmstadt, Germany) and embedded in paraffin. Thin sections of 5 µm were stained with either hematoxylin/eosin (H&E) or Sirius Red (SR). Liver tissue sections were investigated histopathologically by an expert under blinded conditions, with particular attention paid to inflammatory infiltration, hepatocyte damage, bile duct deformation, parasite sections and fibrotic and architectural changes in liver structure. The severity of lesions was graded according to a modified Ishak score as minimal (one foci or less per 10x objective), mild (two to four foci per 10x objective), moderate (five to ten foci per 10x objective), or severe (more than 10 foci per 10x objective) (23). In this study, the modified Ishak scoring system was extended to include an additional histologic feature of parasitic sections. Sites without lesions were referred to as normal (none). Histopathological findings were classified according to lesion appearance, staining and histological features of the liver, with reference to the detailed algorithm of Batts and Ludwig (24). The total amount of collagen in weighed liver fractions was quantified on the basis of colorimetric detection of hydroxyproline using a Quickzyme Total Collagen assay kit (Quickzyme Bioscience, Leiden, The Netherlands) according to the manufacturer´s instructions.

### Quantitative real-time-PCR analysis

At 52 weeks post-infection, total RNA was isolated from snap frozen liver samples (RNeasy Plus Mini Kit, Qiagen, Hilden, Germany) and reversely transcribed into cDNA using High-Capacity cDNA Reverse Transcriptase Kit (ThermoFisher Scientific, Waltham, MA, USA) according to the manufacturer’s instructions. RT-PCR was performed using the following TaqMan Gene Expression Assays: *IL-1β* Mm00434228, *IL-4* Mm00445259, *IL-6* Mm00446190, *IL-10* Mm01288386, *IL-12α* Mm00434169, *IL-13* Mm00434204, *IFN-γ* Mm01168134, *Acta-2* Mm00725412, *Col1-α-2* Mm00483888 (ThermoFisher Scientific, Waltham, MA, USA). Cycling was performed using QuantStudio 3 under the following reaction conditions: 50°C for 2 min followed by 95°C for 10 min, 45 cycles at 95°C for 15 s, and at 60°C for 1 min. Gene expression values were normalized to the endogenous reference gene *gapdh* (Rodent GAPDH control reagent, ThermoFisher Scientific, Waltham, MA, USA) and presented as normalized expression values relative to naive controls.

### Cell preparation

Single-cell suspensions were prepared by passing the spleen through a cell strainer (100 μm). This was followed by phosphate buffered saline (PBS, Merck, Darmstadt, Germany) washing and erythrocyte lysis with RBC lysis buffer (ThermoFisher Scientific, Waltham, MA, USA). Cells were washed twice with PBS and cell numbers were quantified using a CASY TT cell counter (OLS-Omni Life Science).

### Quantification of cytokines

To determine cytokine production, isolated splenocytes were cultured in in RPMI 1640 medium supplemented with 10% FCS, 25mM HEPES and antibiotics, and stimulated for 72 hours at 37°C with a 10 µg/ml *S. mansoni* soluble worm antigen preparation (SWAP) produced in-house. The concentrations of cytokines in the cell-free supernatants were determined using DuoSet ELISA kits (R&D Systems, Minneapolis, Canada) for the detection of IFN-γ, IL-4, IL-13 or IL-10 according to the manufacturer’s instructions.

### Ethical statement

Animal experiments were performed in strict accordance with the regulations of the German Society for Laboratory Animal Science and with the European health guidelines issued by the Federation of Laboratory Animal Science Associations. The protocol was approved by the local committee on animal care and use (7221.3-1.1-036/18-1). Full efforts were made to minimize animal suffering.

### Statistics

Statistical analysis was performed using GraphPad Prism 5.0 (GraphPad Software, La Jolla, CA, USA). Values are expressed as mean + SEM. Differences between groups were analyzed using the Kruskal-Wallis test followed by a Dunn’s *post hoc* test. For all statistical analyses, p values < 0.05 were considered significant. *p < 0.05, **p < 0.01, ***p < 0.001.

## Results

### One-year unisexual infection with male or female schistosomes leads to persistent organ alterations and hepatic inflammation

**Figure 1:**
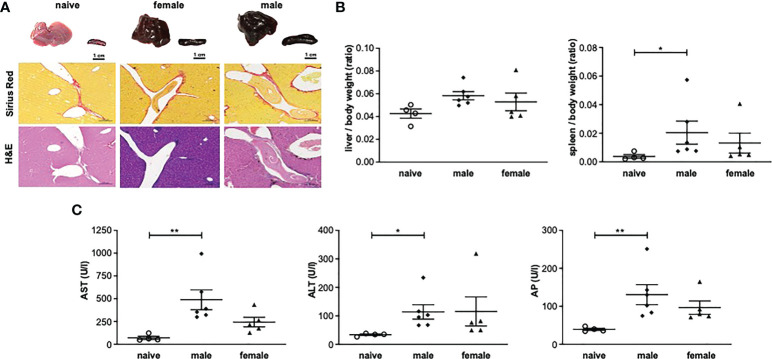
One-year unisexual infection with male or female schistosomes leads to persistent organ alterations and hepatic inflammation. **(A)** Representative images of livers and spleens and of liver sections stained with Sirius red (SR) and hematoxylin/eosin (H&E) (magnification 200-fold). **(B)** Relative organ size of livers and spleens expressed as a ratio to body weight. **(C)** Serum levels of aspartate aminotransferase (AST), alanine aminotransferase (ALT) and alkaline phosphatase (AP) in male-infected, female-infected and uninfected (naive) control group mice (52 weeks post-infection, n=4-6). Data are presented as mean ± SEM. P values < 0.05 were considered statistically significant. *p < 0.05, **p < 0.01.

To analyze the consequences of long-term (52 weeks) unisexual *S. mansoni* infection on the final host organism, pathological organ changes were examined. Macroscopically, the livers of unisexually infected mice appeared dark brown and dull with a smooth surface (**Figure 1A**). Moreover, infection with male or female cercariae resulted in the enlargement of livers and spleens compared with naive mice (uninfected control), as demonstrated by increased liver/body weight and spleen/body weight ratios (**Figure 1B**).

Additional analyses were performed on the liver enzymes aspartate aminotransferase (AST), alanine aminotransferase (ALT) and alkaline phosphatase (AP) as parameters for hepatic inflammation. AST, ALT and AP were significantly increased in the male-infected group compared with naive mice. In the female-infected group, liver enzymes also tended to be increased compared with naive mice (**Figure 1C**).

**Figure 2:**
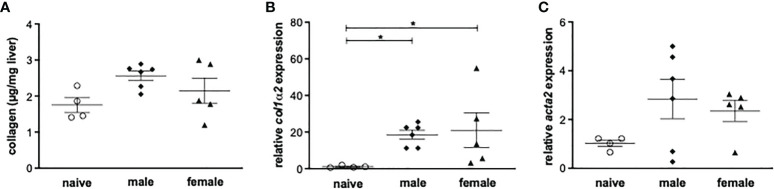
Male or female schistosomes cause liver fibrosis and inflammation following a one-year unisexual infection. **(A)** Collagen deposition in mice livers was quantified by measurement of total collagen (n=4-6) and **(B)** relative gene expression of *col1α2* and **(C)** *acta-2* in livers of male-infected and female-infected mice compared to uninfected (naive) control group mice was determined by real-time PCR (52 weeks post-infection, n=4-6). Data are presented as mean ± SEM. P values < 0.05 were considered statistically significant. *p < 0.05.

Histologically, the livers of mice infected with female or male schistosomes displayed a mild level of focal inflammation, focal lytic necrosis, apoptosis and portal inflammation (**Table 1**). Periportal or peri-septal interface hepatitis (piecemeal necrosis) and fibrosis were present in minimal form in the infected livers. Hepatocellular swelling and cell degeneration with lympho-histiocytic infiltrates are indicators of chronic hepatic damage, as are a dilated portal vein and constipated biliary capillaries, indicating cholestasis. Multiple intravascular worm sections were found. Based on the morphological integrity, it can be assumed that the worms, which were stuck in the liver vasculature, were alive. To investigate these observations more closely, hepatic fibrosis was quantified by measuring hydroxyproline as a marker for collagen deposition (**Figure 2A**). The infected groups exhibited increased collagen levels compared to naive mice. In line with these findings, expression levels of the fibrosis-associated genes *collagen type I alpha 2* (*col1α2*) (**Figure 2B**) were significantly increased in both infected groups, while *alpha actin 2* (*acta2*) also tended to be increased in these groups (**Figure 2C**).

**Table 1:** Histopathological evaluation of liver tissue using a modified Ishak score.

Pathological changes	Naive	Male	Female
Periportal or periseptal interface hepatitis (piecemeal necrosis)	0	1	1
Confluent necrosis	0	0	0
Focal (spotty) lytic necrosis, apoptosis and focal inflammation	1	2.2 ± 0.75	2.2 ± 0.75
Portal inflammation	0	2.2 ± 0.75	2.0 ± 2.0
Fibrosis Stage (SR) staining	0	1	1.4 ± 0.49

### Unisexual infection with male or female *Schistosoma mansoni* induces a non-polarized Th1/Th2 immune response

**Figure 3:**
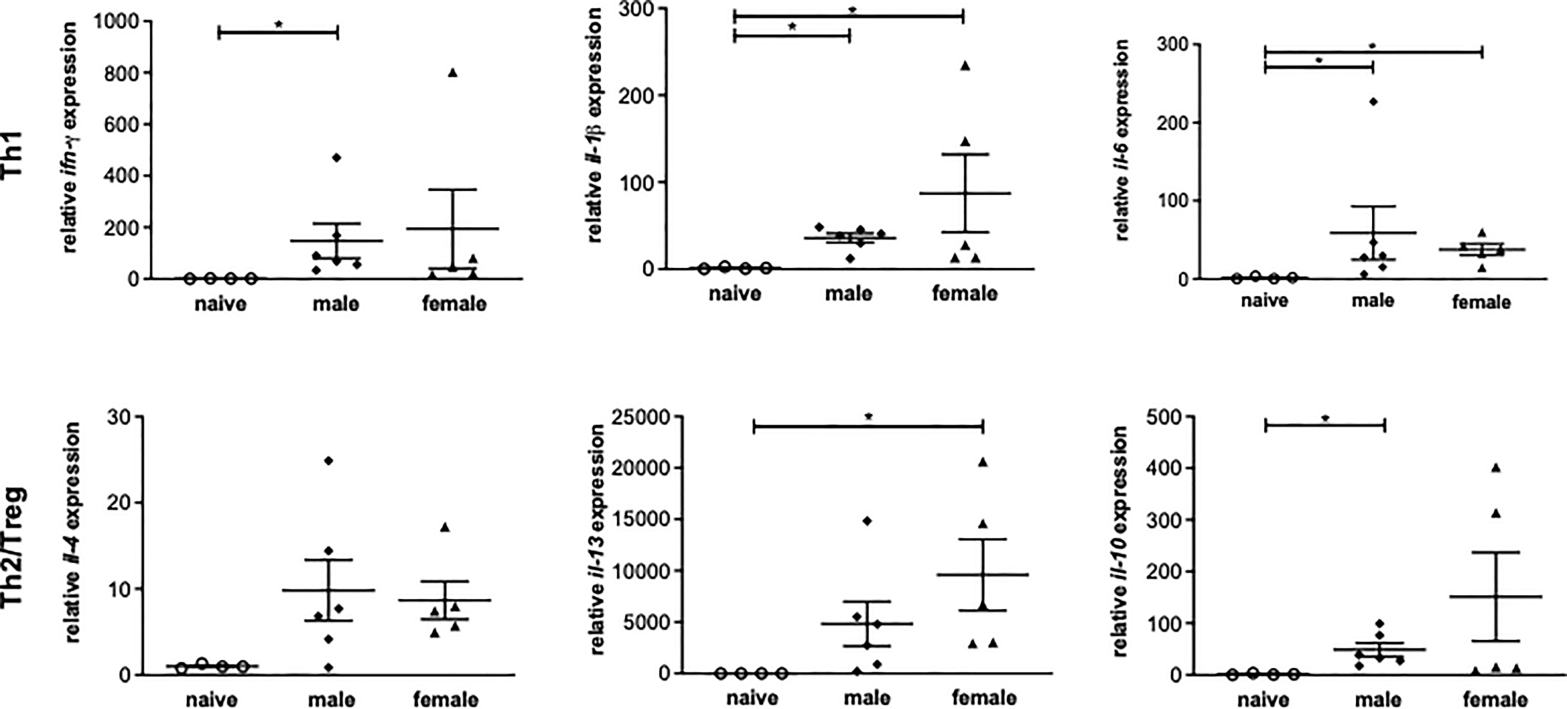
Unisexual infection with male or female *Schistosoma mansoni* induces a non-polarized Th1/Th2 immune response. Relative expression of Th1 and Th2-associated genes in livers of male-infected, female-infected and uninfected (naive) mice (n=4-6, performed in triplicates) was determined by real-time PCR. Data are presented as mean ± SEM. P values < 0.05 were considered statistically significant. *p < 0.05.

To investigate the role of Th1 or Th2 responses in inflammatory events after a yearlong unisexual *S. mansoni* infection, the expression of Th1 and Th2-related genes in liver tissue was analyzed. Following unisexual infection, the gene expression of the Th1 cytokines IFN-γ, IL-1β, IL-6 was upregulated in both the male-infected and female-infected groups compared to naive mice (**Figure 3**). The gene expression levels of the pleotropic Th2 cytokine IL-4 were also upregulated in both groups. IL-13, another important pro-fibrotic Th2 cytokine, was significantly increased in the female-infected group, whereas it was only increased in the male-infected group compared with naive mice. The expression levels of IL-10 were significantly increased in the male-infected group compared to naive mice. In the female-infected group, IL-10 values varied extensively. In summary, infection with exclusively male and with exclusively female schistosomes triggers a strong host immune reaction, generating both a Th1 and a Th2 response.

### Splenocytes isolated from unisexually infected mice produce Th1 and Th2 cytokines following stimulation with adult worm antigen

**Figure 4:**
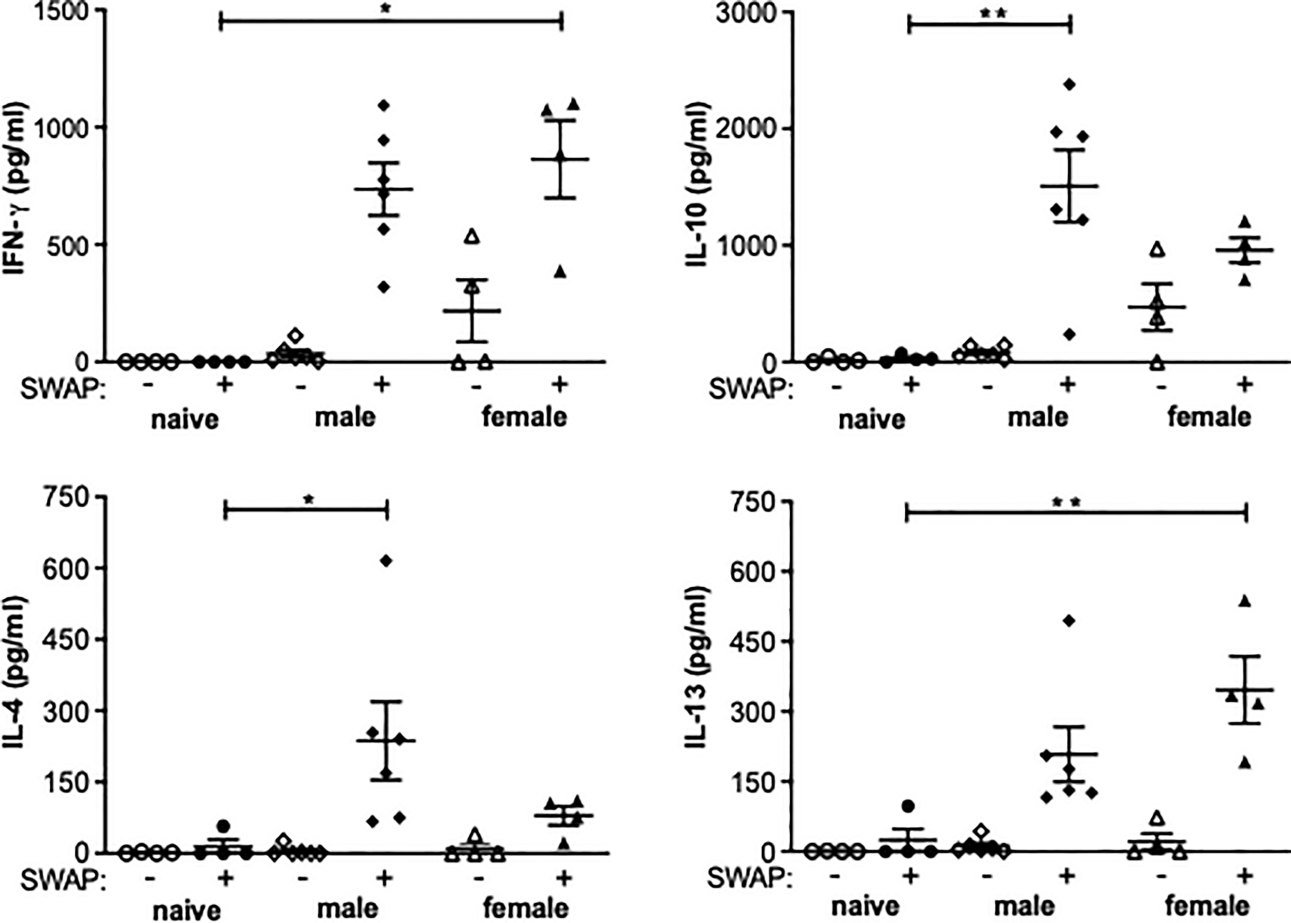
Splenocytes from unisexually infected mice produce Th1 and Th2 cytokines following SWAP stimulation. Splenocytes were isolated from unisexually male-infected, unisexually female-infected and uninfected (naive) control mice and stimulated with 10 µg/ml soluble worm antigen preparation (SWAP). Supernatants were collected after 72 hours and amounts of IFN-γ, IL-4, IL-13 und IL-10 were quantified by ELISA (n=4-6). Data are presented as mean ± SEM from duplicate data. P values < 0.05 were considered statistically significant. *p < 0.05, **p < 0.01.

In both the female-infected and the male-infected groups, increased levels of IFN-y were found in the supernatant of soluble worm/egg antigen (SWAP)-stimulated spleen cells. The increase was significant in the female-infected group compared to naive mice. IL-10 was significantly increased in the male-infected group and slightly increased in the female-infected group. The Th2 cytokines Il-4 and Il-13 were also found in the supernatants of both the male-infected and the female-infected groups stimulated with SWAP. IL-4 was significantly increased in the male-infected group and IL-13 was significantly increased in the female-infected group (**Figure 4**). These data suggest that both male and female schistosomes are capable of eliciting both Th1 and Th2 immune responses, with male schistosomes additionally inducing IL-10 secretion.

## Discussion

The aim of this study was to investigate the effects of a long-term (52 week) unisexual infection with *S. mansoni* in a mouse model, focusing on the predominant immune milieu of the host and the pathology of liver and spleen. We were able to show that long-term unisexual infection with *S. mansoni* leads to significant inflammation of the liver and tissue damage characterized associated with a non-polarized Th1/Th2 response.

In the present study, serum parameters showed increased AST and ALT levels, indicating hepatocyte damage (25). This was unexpected, as in typical schistosomiasis with bisexual infection and oviposition, low hepatocyte damage (26) and consequently low AST and ALT levels have been described (22). Average serum values in the specifications for female NMRI mice provided by the animal supplier are as follows: AST (U/L) = 95 ± 28 and ALT (U/L) = 49 ± 33. This is in line with those measured in the uninfected control group, whereas in the unisexually infected animals, AST and ALT values were outside the normal range. In contrast, the values for AP, a cholestasis marker, were within the normal range in all groups despite the statistical significance of their increase in the male-infected group compared with naive mice. The de-ritis quotient, the ratio between measured serum concentrations of AST and ALT, has long been used to assess hepatocyte damage (27, 28), For obvious reasons, the de-ritis ratio is also known as the aspartate/alanine aminotransaminase (AST/ALT) ratio. In differential diagnostics, the ratio provides information about the type of liver disease present (acute or chronic) as it enables a distinction to be drawn between two types of liver injury: the inflammatory type, in which the quotient is < 1, as occurs in viral hepatitis, or the necrotic type, in which the quotient is > 1, as occurs in alcoholic hepatitis. More recent studies describe a de-ritis ratio of up to 2.0 as a normal value (29). The AST and ALT values measured in this work and the resulting de-ritis quotient (m: 5.1, f: 2.7, n: 2.1) are consistent with the macroscopic diagnosis of inflammatory hepatitis (**Table 1**) caused by long-term unisexual infection, irrespective of worm sex.

In natural infection, oviposition by paired schistosomes in mice starts around 4 to 6 weeks after infection. Since hepatic inflammation is confined to perioval granulomas, the liver parenchyma is mostly unaffected and intact. In the absence of oviposition, liver inflammation occurs in the form of mild focal inflammation, focal lytic necrosis, apoptosis, and portal inflammation to the point of periportal or periseptal interface hepatitis. Upon preparation of the two infected groups, high numbers of live worms were found in the portal vein and the intrahepatic blood vessels, with the male schistosomes more likely to be found in the portal vein and the females deeper in the liver. In this context, it must be taken into account that the maturation of male and female schistosomes depends on different factors. The maturation of male schistosomes progresses independently of females (30, 31) and is more reliant on the immunocompetence of the host than that of females (32). The latter develop in two phases. In the first, approximately four-week post infection phase, females develop independently of males. Subsequently, full maturation into an egg-laying female is dependent on mating with a male worm. In the absence of a male partner, female worms remain in an immature, underdeveloped state (33). This could explain why unpaired female worms were swept deeper into the hepatic vasculature with the bloodstream during the experimental period described.

Although we were not able to adequately quantify the worm burden of both worm sexes, it can be hypothesized that the hepatitis observed in this study may be due to worms trapped in the intrahepatic blood vessels. However, why both worm sexes produce comparable levels of hepatic inflammation despite having predominantly female worms in the liver could not be explained by this hypothesis. Boissier and colleagues demonstrated a significantly greater inflammatory response after unisexual infection with 100 male *Schistosoma mansoni* cercariae (16). They attributed these results to a difference in the quantity of worm antigens due to the underdevelopment of the female worms from unisexual infection compared to the fully developed, significantly larger male worms. In addition, there is an increased amount of vomitus produced by male worms compared to females from unisexual infection. In the present study, we did not find significant differences in the inflammatory response produced by the two worm sexes. In addition, we worked with a threefold increased amount of cercariae in this study. In our own previous (18, 20) and current re-infection studies, we also unisexually infected with 100 cercariae. Accordingly, the amount of worm antigens does not seem to play a major role in the extent of inflammation, either systematically or in relation to the liver. And this is a topic that definitely has to be followed up.

It should also be noted that in the experimental approach described, an important antigenic factor, hemozoin, which is normally produced in large quantities by female worms, appears to be produced less. Adult worms, because of their habitat in the blood, take up nutrients directly through the tegument or by consuming red blood cells (34). Feeding by ingestion of red blood cells, hematophagy, and digestion of hemoglobin results in the formation of toxic heme which must be metabolized into hemozoin. This hemozoin is regurgitated into the blood by the same route due to a lack of anus in the digestive tract of schistosomes and has antigenic potential (35, 36). Unmated female worms not only remain in an underdeveloped stage, they also produce significantly less hemozoin in vomitus than their mated conspecifics. Mated female worms take up approximately 10 times more red blood cells per hour than males (per day males 100 nl and females 900 nl) (34), most likely due to egg production. This is not the case for unisexual infection. Normally, mated female worms appear black compared to male worms due to the high hemozoin content. Underdeveloped unmated females are pale and significantly smaller than males or mated females. Therefore, our study has some limitations. We were able to show that the worms survive unpaired for a very long time, but we failed to show the consequences for the host in a direct comparison between male and female worms, because we actually only looked at adult males and underdeveloped females. Another limiting factor is the lack of quantification of the worm load. The worms cannot be flushed out of the portal vein as by conventional methods. As described above, especially the females are deeply trapped in the hepatic veins. A measurement of CCA or CAA (37) would be feasible. But also, here the quantification of the worm burden would most likely be falsified by the comparison between adult males and underdeveloped females.

Although we were not able to adequately quantify the worm burden of both worm sexes, it can be hypothesized that the hepatitis observed in this study may be due to worms trapped in the intrahepatic blood vessels. However, why both worm sexes produce comparable levels of hepatic inflammation despite having predominantly female worms in the liver cannot be explained by this hypothesis. Boissier and colleagues demonstrated a significantly greater inflammatory response after unisexual infection with 100 male *Schistosoma mansoni* cercariae (16). They attributed these results to a difference in the quantity of worm antigens due to the underdevelopment of the female worms from unisexual infection compared to the fully developed, significantly larger male worms. In the present study, we did not find significant differences in the inflammatory response produced by the two worm sexes. In addition, we worked with a threefold increased amount of cercariae in this study. Interestingly, the clinical picture due to unisexual infection is comparable to our previous and current studies, in which we infected with 50 up to 100 cercariae (18, 20). However, 100 or 300 cercariae in the unisexual infection mouse model represent very artificial infection models that have little in common with the infection expected in nature. In a controlled human *Schistosoma mansoni* model, volunteers were infected with 20 male *S. mansoni* cercariae. Of these, 18% developed severe adverse effects, while the other 82% tolerated the infection well. The authors of this study also emphasize the importance of the infectious dose for the outcome of the infection (38). Consequently, a 300 cercariae infection of a mouse should kill or at least severely impair it. The mice in these studies survived for one year. Therefore, we assume that, in addition to the quantity of worm antigen, other factors determine the clinical picture after unisexual infection such as the genetics of the host. This issue should be addressed in further studies involving a balanced amount of worm antigens and especially low infection studies.

Soluble egg antigens of schistosome eggs are well-understood immune modulators (12, 39). With the onset of egg deposition in a natural infection, there is a shift from a Th1 to a Th2 response (40). However, a study by Oliveira Fraga et al. demonstrated that, *via* CD4^+^ T cells, the worms themselves are capable of indirectly inducing a Th2 immune response through the production of IL-4 (41), an important mediator in Th2 immunity (42). Another important cytokine detected at increased expression levels in the livers of the male-infected group compared with the naive group is regulatory IL-10. IL-10 inhibits the production of IL-12, which plays an important role in the formation of a Th1 response (43). Together with IL-4, it also moderates the development of the potentially life-threatening inflammation for the infected host that can be triggered by a Th1 response (44). The role of IL-4 and IL-13 in the granuloma formation and fibrosis associated with schistosomiasis was the subject of a study by Chiaramonte et al. (45). In that study, IL-4 was shown to play a redundant role in granuloma formation, as evidenced by the ability of IL-4 knockout mice to form perioval granulomas. At the same time, IL-13 was shown to be the dominant cytokine in fibrosis. IL-13 knockout mice displayed reduced fibrosis as a result of the reduced expression of procollagen I and III in fibroblasts. In IL-4 knockout mice, a much smaller reduction in fibrosis occurred. In a similar study by Fallon et al., IL-4 and IL-13 knockout mice displayed a Th1 response to SWAP stimulation of isolated splenocytes after infection with *S. mansoni* (46). In other words, without IL-4 and IL-13, there would be no Th2 response and no fibrosis.

The cytokine picture often drawn for experimental schistosomiasis describes an early transient Th1 response with a Th2 switch at the onset of oviposition which then transitions to a Th2/Treg during chronic disease progression (47). It is hypothesized that the suppressed Th1 and sustained Th2/Treg response typical of chronic schistosomiasis is driven by parasite eggs. Our results suggest that worms alone, independent of egg production, are capable of eliciting a Th2 response. The question of the role of the IL-10 measured in the male-infected group remains open.

After 52 weeks of infection, live worms, both male and female, could still be observed. This suggests that the worms persist in the host until a suitable worm partner (worm of the opposite sex) arrives to mate with them. This assumption is supported by another study (48), in which a first unisexual infection with male *Schistosoma japonicum* was followed by a second unisexual infection with females, after which mating was indeed detected.

In summary, unisexual schistosomes survive in mice for at least one year. They cause visible and measurable hepatitis during infection associated with a non-polarized Th1/Th2 response. However, the immature stage of development of the female worms as a comparison group to the mature adult worms must be considered here. The extent to which unpaired worms would still be able to mate in the presence of a suitable partner after a year is uncertain. Also, the question arises as to whether and how an emerging immunomodulatory effect brought about by parasite eggs would affect the host this long after the onset of unisexual infection.

## Data availability statement

The original contributions presented in the study are included in the article/supplementary material. Further inquiries can be directed to the corresponding author.

## Ethics statement

The animal study was reviewed and approved by local committee on animal care and use (7221.3-1.1-036/18-1).

## Author contributions

Conceptualization, MS, CR, ER. Formal analysis, MS, CR, NK, FW. Investigation, MS, CR, FW, AR, NK. Data maintenance, MS and CR. Writing - preparation of first draft, MS and CR. Writing - review and editing, MS, FW, ER. Visualization, MS. Supervision, MS, ER. All authors contributed to the article and approved the submitted version.

## Acknowledgments

The skillful technical support of Daniel Wolter and Mohamed Elhensheri (Department of Oral, Maxillofacial, and Plastic Surgery, University Medical Center Rostock) is gratefully acknowledged. The work was funded by the European Social Fund project Card-ii-omics (ESF/14-BM-A55-0037/16).

## Conflict of interest

The authors declare that the research was conducted in the absence of any commercial or financial relationships that could be construed as a potential conflict of interest.

## Publisher’s note

All claims expressed in this article are solely those of the authors and do not necessarily represent those of their affiliated organizations, or those of the publisher, the editors and the reviewers. Any product that may be evaluated in this article, or claim that may be made by its manufacturer, is not guaranteed or endorsed by the publisher.
